# Prognostic role of myoferlin expression in patients with clear cell renal cell carcinoma

**DOI:** 10.18632/oncotarget.21645

**Published:** 2017-10-06

**Authors:** Dae Hyun Song, Gyung Hyuck Ko, Jeong Hee Lee, Jong Sil Lee, Jung Wook Yang, Min Hye Kim, Hyo Jung An, Myoung Hee Kang, Kyung Nyeo Jeon, Dong Chul Kim

**Affiliations:** ^1^ Gyeongsang National University School of Medicine, Jinju, South Korea; ^2^ Gyeongsang Institute of Health Science, Gyeongsang National University, Jinju, South Korea; ^3^ Department of Pathology, Gyeongsang National University Hospital, Jinju, South Korea; ^4^ Department of Pathology, Gyeongsang National University Changwon Hospital, Changwon, South Korea; ^5^ Department of Internal Medicine, Gyeongsang National University Changwon Hospital, Changwon, South Korea; ^6^ Department of Radiology, Gyeongsang National University Changwon Hospital, Changwon, South Korea

**Keywords:** clear cell renal cell carcinoma, myoferlin, prognosis, disease-free survival, immunohistochemistry

## Abstract

**Objectives:**

In patients with cancer, myoferlin protein hyperexpression has been correlated with poor patient prognosis. Here, we evaluated myoferlin expression in patients with clear cell renal cell carcinoma (ccRCC) and investigated the prognostic significance of myoferlin expression in these patients.

**Materials and Methods:**

One hundred and fifty-two patients with ccRCC who underwent treatment at Gyeongsang National University Hospital, Korea, between January 2000 and December 2009 were enrolled. Immunohistochemical analysis was performed on tissue microarray blocks produced from surgical specimens. Surgical specimen cancerous cells were graded as showing myoferlin hyperexpression or hypoexpression by comparison with intratumoral endothelial cells. Disease-free survival was evaluated using Kaplan-Meier analysis. Cox regression analysis was used to determine the relationships between myoferlin expression levels, risk factors, and prognosis.

**Results:**

Seventy-one of 304 cores exhibited myoferlin hyperexpression. T stage was not associated with myoferlin hyperexpression, whereas a high Fuhrman nuclear grade was significantly associated with myoferlin hyperexpression. Kaplan-Meier analysis revealed that patients with T stage >2, Fuhrman nuclear grade >2, and those with myoferlin hyperexpression had poorer disease-free survival compared to those with lower T stage, lower Fuhrman nuclear grade, and myoferlin hypoexpression (all p <0.001). Furthermore, myoferlin hyperexpression was significantly associated with disease-free survival on Cox regression analysis (hazard ratio, 4.604; 95% confidence interval, 1.893–11.199; p = 0.001).

**Conclusion:**

Myoferlin expression could be a potential prognosticator in patients with ccRCC, and might be a useful marker for oncologic surveillance in such patients.

## INTRODUCTION

Clear cell renal cell carcinoma (ccRCC) accounts for 70–80% of RCCs [1], and ∼20–30% of these patients progress to an advanced stage (recurrence or distant metastasis) after surgery [2]. The frequency of postoperative radiologic surveillance, which consists of chest and abdominal radiologic imaging, is determined by a patient’s 5-year risk for recurrence or distant metastases [3, 4]. The tumor-node-metastasis staging of the American Joint Committee on Cancer (AJCC) [5] and Fuhrman nuclear grade [6] are well established predictive risk factors for recurrence. However, to ensure that treatment for patients with ccRCC patients is efficacious, additional reliable prognosticators are needed.

Myoferlin protein expression has been recently associated with poor prognosis in patients with cancer [7–9]. The 6-membered Ferlin family are mammalian proteins that share homology with the Fer-1 family of *Caenorhabditis Elegans* [10] and include myoferlin, dysferlin, otoferlin, fer1L4, fer1L5, and fer1L6. Fer-1 defects have been associated with infertility due to the abnormal fusion of cellular membranes in developing sperm [11]. Ferlins have also been implicated in calcium-sensing, cellular repair, vesicle trafficking, and membrane fusion in skeletal muscle [12]. In normal embryonic development or muscle regeneration, myoferlin is highly expressed during myoblast fusion [12]. However, the physiological function(s) of myoferlin have not been fully elucidated.

Myoferlin overexpression has been noted in gastric, thyroid, breast, colorectal, hepatic, pancreatic, ovarian, cervical, and endometrial cancers [13]. Furthermore, in MDA-MB-231 human breast cancer cells, myoferlin depletion induced a mesenchymal-to-epithelial transition, *in vitro*, and reduced tumoral proliferation and invasion, *in vivo* [14, 15]. Leung *et al.* showed tumorigenetic role of myoferlin [16]. According to recent clinical studies, patients with pancreatic or oropharyngeal squamous cell carcinoma that showed myoferlin hyperexpression had poorer survival outcomes [9, 17].

Here, we investigated myoferlin expression in patients with ccRCC and evaluated the prognostic significance of myoferlin expression in these patients.

## RESULTS

### Clinicopathological data

**Figure 1:**
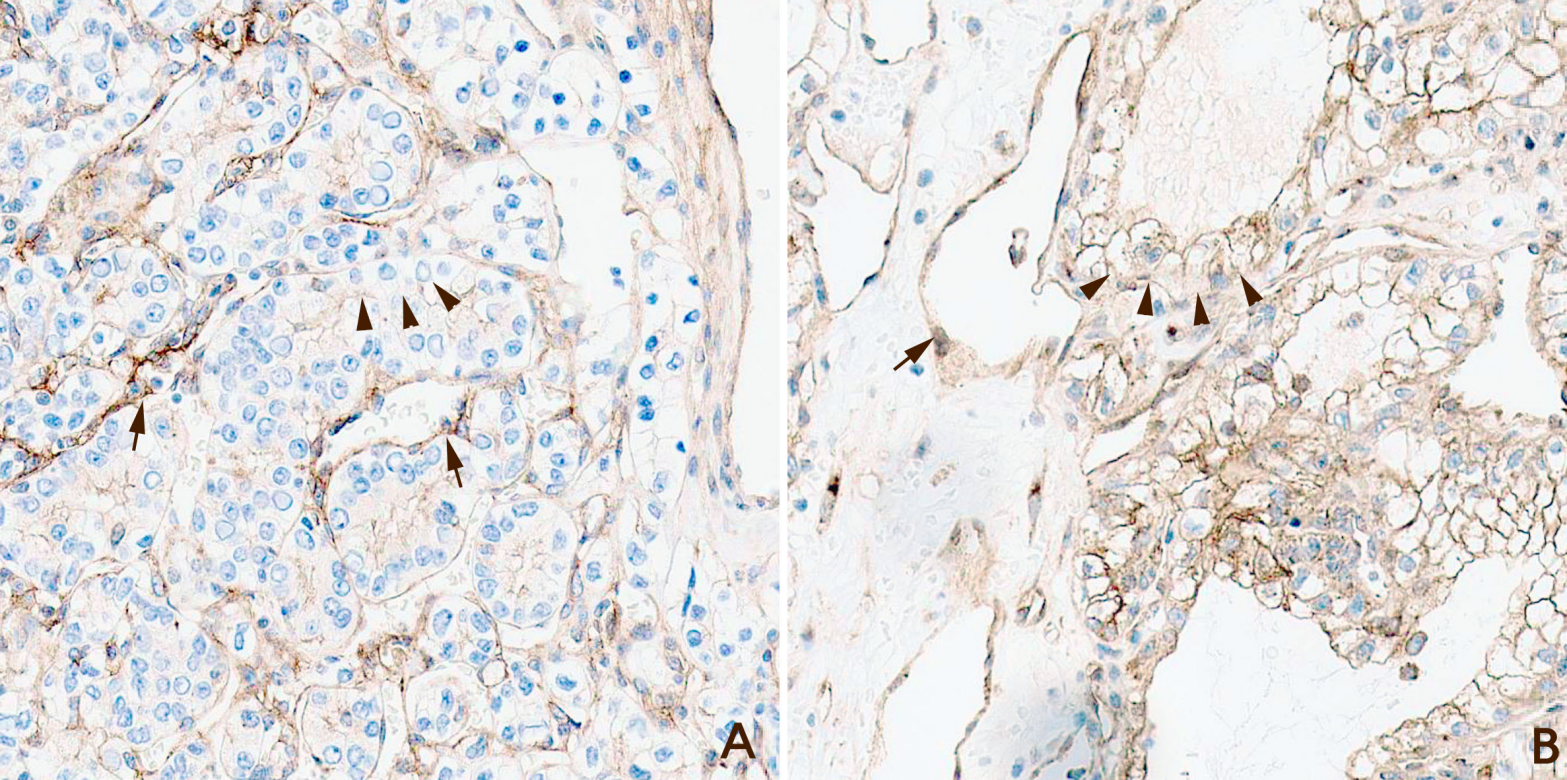
Examples of myoferlin staining grades **(A)** Myoferlin hypoexpression. Clear cell renal cell carcinoma (ccRCC) cells revealed weaker expression compared with capillary endothelial cells (arrow, endothelial cells; arrow head, ccRCC cells; Magnification x200). **(B)** Myoferlin hyperexpression. Clear cell renal cell carcinoma (ccRCC) cells exhibited stronger expression compared with capillary endothelial cells (arrow, endothelial cells; arrow head, ccRCC cells; Magnification x200).

The clinicopathological data of patients with ccRCC (n =152) are summarized in Table 1. There were 115 (75.7%), 12 (7.9%), 23 (15.1%), and 2 (1.3%) patients with T stage 1, 2, 3, and 4 disease, respectively. Twenty-five patients had progression of diseases, Of which nine patients exhibited metastatic ccRCC of the lung. And 6 patients showed metastasis to multiple organs, such as the lung, bone and/or brain. The majority of patients had Fuhrman nuclear grade 2 tumors. Of the 304 cores evaluated, 71 exhibited myoferlin hyperexpression (Hyper-MYOF), and 233 had myoferlin hypoexpression (Hypo-MYOF) (Figure 1A and 1B).

**Table 1:** Clinicopathological information of 152 clear cell renal cell carcinoma(RCC) patients

	Variable		Value (percentage)
	Patients number		152
	Cores number of TMA		304
Clinical information	Age [range]		mean 59.9 [32–83]
	Sex (Male/Female)		109/43 patients
	Advanced RCC	Lung metastasis	9 patients
		Multiple metastasis	6 patients
		Bone metastasis	4 patients
		Brain metastasis	2 patients
		Liver metastasis	1 patients
		Local recurrence	3 patients
	Follow-up period		Mean 4.33 years
Patologic information	T stage	1a	91 (59.9%) patients
		1b	24 (15.8%) patients
		2a	9 (5.9%) patients
		2b	3 (2.0%) patients
		3a	21 (13.8%) patients
		3b	2 (1.3%) patients
		4	2 (1.3%) patients
	Fuhrman grade	1	26 (17.1%) patients
		2	102 (67.1%) patients
		3	19 (12.5%) patients
		4	5 (3.3%) patients
	Myoferlin	Hyperexpression	71 (23.4%) cores
		Hypoexpression^*^	233 (76.6%) cores
Total			152 patients 304 cores

*, hypo-expression contains negative expression.

### Correlation of myoferlin expression with T stage and Fuhrman nuclear grade in patients with ccRCC

The relationships between myoferlin expression and T stage are summarized in Table 2. There were no significant differences in the incidence of Hyper-MYFO among patients with T stage 1 and those with T stage ≥2 (Chi squared, p = 0.240). The relationships between myoferlin expression and Fuhrman nuclear grade are summarized in Table 3. A Fuhrman nuclear grade ≥3 was significantly associated with the incidence of Hyper-MYOF (Chi squared, p < 0.001).

**Table 2:** Correlation between myoferlin expression, T stage and Fuhrman nuclear grade in clear cell renal cell carcinomas

		Myoferlin expression		Total	P value
		Hypo*	Hyper		
**T stage**	1	180	50	230	P=0.240
	≧2	53	21	74	
**Fuhrman nuclear grade**	1,2	205	50	255	**P<0.001**
	3,4	28	21	49	
Total		233	71	304	

*, hypo-expression contains negative expression.

**Table 3:** Multivariate analyses of recurrent-free survival in 152 clear cell renal cell carcinoma patients

Variable		Disease-free survival			Disease-specific survival		
		HR	95% CI	p value	HR	95% CI	P value
Age	**<59 vs.** ≧**59**	3.497	1.372 - 8.915	**0.009**	0.945	0.453-1.972	0.880
Sex	**male vs. female**	1.339	0.428- 4.191	0.617	0.221	0.084-0.585	**0.002**
T stage	**1 vs. 2∼4**	22.313	7.719-64.494	**<0.001**	33.420	10.938-102.109	**<0.001**
Fuhrman grade	**1,2 vs. 3,4**	2.997	1.270-7.070	**0.012**	4.009	1.694-9.487	**0.002**
Myoferlin	**hypo vs. hyper**	4.604	1.893-11.199	**0.001**	1.240	0.490-3.135	0.650

### The significance of Hyper-MYOF on survival in patients with ccRCC

**Figure 2:**
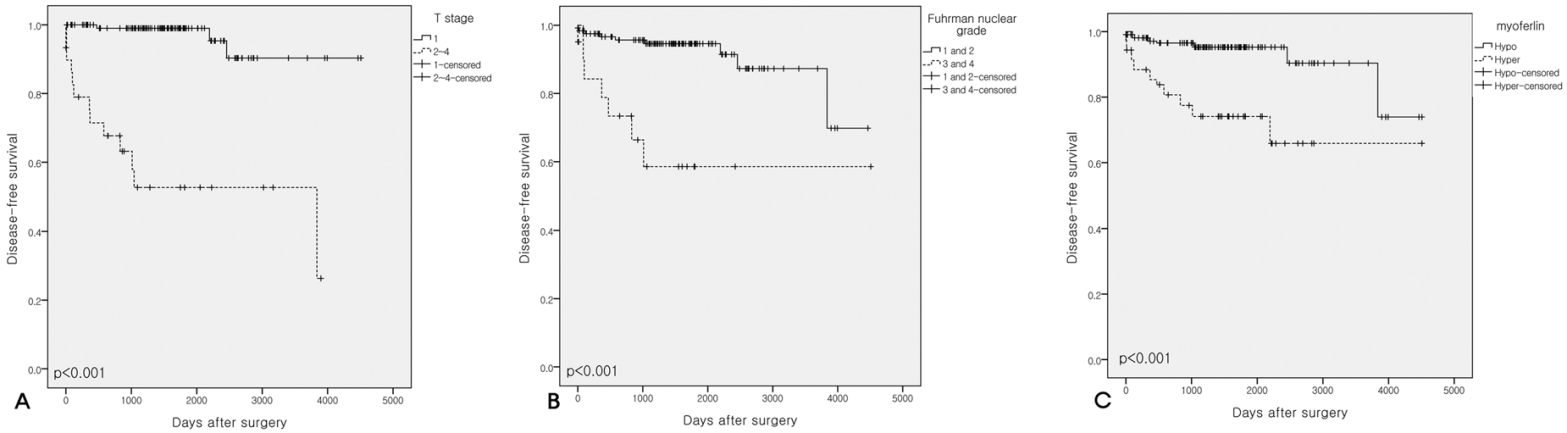
Kaplan-Meier survival curves **(A)** Disease-free survival according to T stage. **(B)** Disease-free survival according to Fuhrman nuclear grade. **(C)** Disease-free survival according to myoferlin expression levels. High T stage, high Fuhrman nuclear grade, and myoferlin hyperexpression were all significantly associated with poorer disease-free survival.

According to Kaplan-Meier analysis, patients with Hyper-MYOF, high T stage (≥2), or high Fuhrman nuclear grade (≥3) had poorer disease-free survival (DFS, p <0.001) compared to patients with Hypo-MYOF (Figure 2A-2C). Cox proportional hazards regression analysis revealed that age ≥59 years (hazard ratio [HR], 3.497; 95% confidence interval [CI], 1.372–8.915; p = 0.009), T stage ≥2 (HR, 2.313; 95% CI, 7.719–64.494; p <0.001), Fuhrman nuclear grade ≥3 (HR, 2.997; 95% CI, 1.270–7.070; p = 0.012), and Hyper-MYOF (HR, 4.604; 95% CI, 1.893–11.199; p <0.001) were significantly associated with DFS (Table 3). Hyper-MYOF was not significantly associated with disease-specific survival (DSS, p = 0.650; Table 4).

**Table 4:** Clinicopathological information of advanced renal cell carcinoma patients with T1 stage tumor

Patient number	Sex/age	T stage	Furhman nuclear grade	Myoferlin expression	Follow up duration to recurrence (years)	Recurrence
No.1	M/64	1a	2	Hyper	6	Local recurrence
No.2	M/67	1a	3	Hyper	3.8	Lung meta*
No.3	M/71	1b	3	Hypo	7.6	Lung meta
No.4	F/78	1a	3	Hypo	3.4	Bone meta
No.5	M/72	1a	1	Hypo	6.8	Bone meta

*, meta means metastasis

## DISCUSSION

In the present study, ccRCC patients with Hyper-MYOF had significant poorer DFS compared to those with Hypo-MYOF. However, Hyper-MYOF was not associated with DSS in patients with ccRCC. This is the first study to demonstrate an association between high myoferlin expression and prognosis in patients with ccRCC. Furthermore, a high Fuhrman grade was associated with Hyper-MYOF, whereas T stage was not associated with myoferlin hyperexpression.

Here, we found that myoferlin might be a valuable prognostic marker in patients with ccRCC. Particularly, Hyper-MYOF had greater significance than the Fuhrman nuclear grade at predicting DFS. Furthermore, although, T stage is considered a valuable prognostic factor, in the present study, 5 patients with advanced T1 stage primary ccRCC, of whom 3 had Fuhrman nuclear grade 3 (high) and 2 had Fuhrman nuclear grade 2 (low), also had Hyper-MYOF (Table 4). Therefore, we suggest that even in patients with a low T stage, a high Fuhrman grade or Hyper-MYOF could indicate those that should be followed-up more carefully.

There are some limitations to the present study. For example, Hyper-MYOF was not associated with DSS. The lack of effect on DSS could be because of biases. For example, patients with advanced ccRCC are treated using different therapies such as the tyrosine kinase inhibitors (e.g., sunitinib or pazopanib) and inhibitors of the mammalian target of rapamycin. In addition, the initiation of treatment varied among the patients. Such differences could confound the data. Therefore, clinical evaluations considering such factors are needed to validate the significance of myoferlin expression on prognosis.

In conclusion, myoferlin overexpression had an adverse prognostic role in patients with ccRCC and might be a valuable biomarker for ccRCC oncologic surveillance. Future studies to validate the role of myoferlin in the prognosis of such patients are warranted.

## MATERIALS AND METHODS

### Patients

The clinicopathological data of patients with ccRCC patients who underwent treatment at Gyeongsang National University Hospital, Jinju, Korea between January 2000 and December 2009 were obtained by reviewing clinical electronic charts, retrospectively. One-hundred and fifty-two patients with ccRCC were enrolled. All patients had undergone nephrectomy. The diagnosis was histopathologically confirmed by 2 experienced pathologists. All tumors were staged using the AJCC 7^th^ system [5].

DFS was defined as the duration from the date of surgery to the date of tumor recurrence. DSS was defined as the duration from the date of surgery to date of death, which was mostly due to ccRCC. Advanced ccRCC with recurrence was diagnosed histopathologically via surgical biopsy or by radiography. A radiological diagnosis of lung metastasis was made when solitary or multiple spherical nodule(s) could be seen on chest computed tomography (CT) scans. Multiple lung metastases were variable in size and distributed throughout the pulmonary lobes. A radiological diagnosis of bone metastasis was made when solid enhancing masses or bone destruction became newly apparent on CT scans during follow-up. In all patients with radiologically suspected metastatic lesions, a final diagnosis was confirmed via CT-guided percutaneous biopsy and/or by combined positron emission tomography/noncontrast CT or scintigraphy [18, 19]. This study was approved by the Institutional Review Board of Gyeongsang National University Hospital (GNUH-2015-12-001) and was conducted in accordance with the principles embodied in the Declaration of Helsinki.

### Tissue microarray

Nephrectomy specimens were fixed in buffered neutral formalin (20%) overnight. Surgical samples were examined grossly and then embedded in paraffin blocks. Two representative glass slides from matched paraffin blocks were selected under microscopic review (Nikon Eclipse Ni). Two 2-mm tissue cores were obtained from each representative paraffin block that has representative Fuhrman nuclear grading at intratumoral area. and transplanted to new recipient tissue microarray blocks.

### Immunohistochemical analysis

Immunohistochemical staining was performed using a primary myoferlin antibody (1:100 dilution; 7D6, Abcam, UK) as described previously [7]. The endothelial cells of the intratumoral vessels were used as a positive control. Slides from tissue microarray cores were graded by two experienced pathologists. If ccRCC cells exhibited stronger myoferlin expression compared with the membrane or cytoplasm of the endothelial cells then staining was graded as Hyper-MYOF. Similarly, if myoferlin expression was weaker than that of endothelial cells staining was graded as Hypo-MYOF. Negative expressed cases were contatined in Hypo-MYOF group. If the tumor cells showed heterogeneity of myoferlin in a core, representative value of the core was decided as a value of majority of tumor cells (more than 50%). Myoferlin expression of endothelial cells was reported in previous study [20]. And intratumoral endothelial cells were used as control of myoferlin expression.

### Statistical analyses

Correlations between variables were determined using the Chi squared test. Cumulative survival times were evaluated using the Kaplan-Meier method and the log-rank test. Multivariate analysis using the Cox proportional hazards regression model was conducted to compare variables. A p-value of <0.05 was considered statistically significant. SPSS ver. 24.0 (SPSS Inc., Chicago, IL, USA) was used for all statistical analyses.
